# Abuse of Remedial Agents

**Published:** 1856-11

**Authors:** J. G. Westmoreland

**Affiliations:** Atlanta, Georgia


### ARTICLE IV.

*Abuse of Remedial Agents.* By J. G. WESTMORELAND, M. D., of Atlanta, Georgia.

The abuse of the most valuable remedies, as well as the most nutritious articles of diet, has always a contrary effect from that observed in their proper and rational use. The gormandizer finds anything but strength and activity of mind and body to result from the enormous quantity of food required to satiate his voracious appetite. Food and medicine prove a blessing, when properly used, but the most destroying curse when given out of place, or in excessive quantities.

The impression prevails, in the community generally, that medicine is as essential to the sick, as food to the hungry; and that to derive the required benefit from physic, it must *work*, must *operate*.

Now, to those not informed on Medical subjects, knowing nothing of the nature and *modus operandi* of remedies, all remedial means seem unpromising, unless they augment perceptibly some of the prominent physiological actions, producing catharsis, emesis, diaphoresis, or some such effect. Hence, those remedies making the most decided impressions in this way are the most popular ones. It is not a very easy matter for one unaccustomed to their use to perceive the impression made on the disease itself with medicine. A violent action may be exerted on the organs, and when it has subsided the invalid may feel such comparative ease, that a superficial observer, and the patient himself, may conclude the disease is controlled, when, in fact, no impression upon it has been made. Cathartics, as a class, of all others, are most abused in this way. It is an extensive class, comprising articles calculated to produce any degree of action, from the natural healthy discharge to the most drastic watery evacuation. Their effects are visible; and, in their action it may be truly said, the *physic* has *operated.* Charlatans, availing themselves of the advantages derivable from this popular feeling, flood the world with "Mild Aperient," "Anti-billious," "Chologogue," and "Cathartic pills;" and to secure the patronage of mineral fearers endorse upon the box, "purely vegetable." The better to effect this wholesale pilling, presses are hired to puff box after box down the throat of their unsuspecting neighbor.

The clergyman is presented with a box of pills, made of the most familiar ingredients. These, after producing the usual aperient effect of medicines of the class, are, from the mystery thrown around their composition, thought new and valuable, and proclaimed a useful remedy, by certificates and otherwise.

The statesman, as well as the preacher, is supposed to know all about everything; therefore, his testimony, on a subject about which he knows literally nothing, is obtained, greatly to the advantage, not of the sick, but of him, who, for pecuniary gain, deceives his neighbor, and sells him at an exorbitant price, that which, if known, he would not desire to accept as a present. And this is not all the evil that comes of cathartics. When I say evil I mean no disparagement to the claims of this valuable class of remedies; for invaluable are they to the therapeutist.

But, to carry out the sentiment with which this article was commenced, great injury may result from the improper use of good things.

Such is the case with this class of medicine, not only when under the control of merciless quacks, but when prescribed by those governed by high and honorable motives in their use. In despite of all our philosophy and sense of propriety, we, of the medical profession, sometimes do that for which we can give no satisfactory reason to ourselves nor to any one else.

Perhaps habit or precedent often controls our action, and leads to performances not tolerated by reason. Sometimes, too, when a proclivity is observed in those whom it is our *interest* to please, we are inclined to cater to the prejudices of such patients.

These influences imperceptibly creep upon us, and often before we are aware of it, a habit is formed, for which we can give no satisfactory account. A patient is seen—he says, "I'm costive," "bound in my bowels," "have pain in my belly," &c. Take oil, pills, calomel, or something of the kind, to "purge the bowels," is often the direction of the physician; without weighing the probabilities of benefit, or the cause of distress. The patient and doctor are satisfied when the organ in pain is acted upon; whether "for weal or for woe," however, neither has any certainty. Or, when it seems desirable to make a less common prescription, a compound pill, powder or bolus is prepared, incorporating one or more cathartic articles, and given as a nameless prescription of "medicine." The mucous surface is irritated, the peristaltic action increased, the medicine "*works*," the patient is content, and the doctor too often satisfied.

All this would be no great evil, seeing no particular mischief is done, were it not that occasionally such articles are contra-indicated, and their application places the sufferer beyond the reach of remedies.

This more frequently occurs on account of the alledged universal application of cathartics in disease. In diarrhœa, whether, from relaxation or enteric irritation or inflammation, loud calls are made for "medicine" to remove more thoroughly the offensive fœcal accumulation, which is supposed by the patient, and sometimes the Doctor too, to be the cause of the affection. Pills are repeated again and again—still the fetor and discoloration are prominent characteristics of the discharges. And it is remarkable that it does not once enter their minds that the supply of offensive matters is kept up by the very means intended for their removal. A like mistake prevails with some persons affected with habitual costiveness. Some of the *Indian-antibillious-aperient-purely-vegetable* pills, which are sounded by newspapers as the *sine qua non* in constipation, have been taken; and the bowels were actually moved by them! Yes, nearly as well as they would have been by a dose of magnesia, salts or rhubarb!! The additional charm of secrecy and humbuggery has so attracted the constipated epicure that he fancies his box of pills the only safeguard of his life!

Aside from such whims indulged by the people, the system of indiscriminate purging and "physicking" adopted by physicians themselves, is, no doubt, one of the main supporters of the faith that is professed in the anti-physic, sugar-pill or homœopathic doctrine. The sick, in many cases, do as well with nothing at all as with irritating and depressing remedies. Mild cases of disease, similar in every respect, are treated with improperly applied violent medicine, and with expectant remedies. The latter generally makes the most favorable impression; and, although a proper selection, and discriminating use of remedies might produce better results, the idea of operating medicine is discarded, and that which seems less offensive is substituted.

If active medicines were used properly, and only when required, their control over disease would be more obvious, and people and physicians would properly appreciate their value, and one of the main props to humbuggery and charlatanry be removed.

